# Superior efficacy of immunotherapy‐based combinations over monotherapy for *EGFR*‐mutant non‐small cell lung cancer acquired resistance to EGFR‐TKIs

**DOI:** 10.1111/1759-7714.13689

**Published:** 2020-10-19

**Authors:** Lu Yang, Xuezhi Hao, Xingsheng Hu, Zhijie Wang, Ke Yang, Yuling Mi, Yaning Yang, Haiyan Xu, Guangjian Yang, Yan Wang

**Affiliations:** ^1^ Department of Medical Oncology National Cancer Center/National Clinical Research Center for Cancer/Cancer Hospital, Chinese Academy of Medical Sciences and Peking Union Medical College Beijing China; ^2^ Department of Medical Oncology Cancer Hospital of Huanxing Chaoyang District Beijing Beijing China; ^3^ Department of Medical Oncology Chaoyang Sanhuan Cancer Hospital Beijing China; ^4^ Department of Comprehensive Oncology National Cancer Center/National Clinical Research Center for Cancer/Cancer Hospital, Chinese Academy of Medical Sciences and Peking Union Medical College Beijing China

**Keywords:** Progression‐free survival (PFS), EGFR positive, immunotherapy, non‐small‐cell lung cancer (NSCLC), overall response rate (ORR)

## Abstract

**Background:**

While prospective clinical studies on immunotherapy in epidermal growth factor receptor (EGFR) mutant non‐small‐cell lung cancer (NSCLC) with acquired resistance to EGFR tyrosine kinase inhibitors (TKIs) are ongoing, this study aimed to investigate the outcomes of immunotherapy combinations in such a population in a real‐world setting.

**Methods:**

The clinical data of pretreated *EGFR*‐mutated NSCLC patients who acquired EGFR‐TKI resistance and received immunotherapy were retrospectively analyzed in this study. Progression‐free survival (PFS) was assessed using the Kaplan‐Meier log‐rank test, and univariate and multivariate analysis were performed.

**Results:**

A total of 31 patients were analyzed in this study. A total of 25 (80.6%) patients received combination immunotherapy. In the univariate analysis, patients who received combination immunotherapy seemingly acquired longer PFS than those who received monotherapy, although there was no significant difference (3.42 months vs. 1.61; *P* = 0.078; hazard ratio (HR) 0.43, 95% CI: 0.16–1.13). Patients who received antiangiogenic drugs prior to immunotherapy acquired better PFS (3.42 months vs. 1.58; *P* = 0.027; HR 0.37, 95% CI: 0.15–0.93), while patients with liver metastasis had inferior PFS (2.04 months vs. 3.42; *P* = 0.031; HR 2.83, 95% CI: 1.05–7.60). Furthermore, multivariate analysis confirmed that the above three factors had independent prognostic value.

**Conclusions:**

The study revealed that immunotherapy combinations are better choices than single‐agent regimens in previously treated and *EGFR*‐mutant NSCLC patients with progressive disease. In addition, antiangiogenic drugs administered before immunotherapy might be a favorable prognostic factor, while liver metastasis was associated with a short PFS in this setting. In future, more robust and prospective clinical trial results are expected to guide clinical practice.

**Key points:**

**Significant study findings**

- Immunotherapy‐based combination therapies are better choices than single‐agent regimens in heavily treated *EGFR*‐mutant NSCLC patients.

**What this study adds**

- Patients without liver metastasis and with prior antiangiogenic drugs obtained more benefit from immunotherapy in this setting.

## Graphical abstract

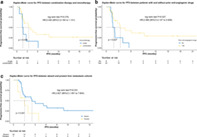

Immunotherapy‐based combination therapies are better choices than single‐agent regimens in heavily treated *EGFR*‐mutant NSCLC patients. Patients without liver metastasis who have previously received antiangiogenic drugs obtain more benefit from immunotherapy in this setting.

## Introduction

Non‐small‐cell lung cancer (NSCLC) accounts for approximately 80% of lung cancer cases and is one of the most common causes of cancer‐associated deaths worldwide.^1^, ^2^ Epidermal growth factor receptor (*EGFR*) gene mutation is the most common genetic alteration driving NSCLC.^3^, ^4^ EGFR tyrosine kinase inhibitors (TKIs), including gefitinib, erlotinib, icotinib, afatinib, dacomitinib and osimertinib, have been confirmed to be effective and well tolerated as first‐line therapy.^5^, ^6^, ^7^, ^8^, ^9^, ^10^ However, acquired resistance inevitably occurs in advanced NSCLC patients, despite an initial dramatic response to EGFR‐TKIs. At present, chemotherapy is still the main treatment for patients who fail to respond to first‐line EGFR‐TKIs without acquired T790M mutation and acquired resistance to third‐line EGFR‐TKIs.^11^

Immunotherapy has become a cornerstone of management in advanced NSCLC without driver gene mutations.^12^, ^13^, ^14^ Nevertheless, it has not showed efficacy in patients with *EGFR*‐mutated tumors. Many patients with *EGFR* mutations who received anti‐programmed death 1 (PD‐1) antibodies or anti‐PD‐L1 antibodies did not achieve a favorable outcome in multiple clinical and retrospective studies.^13^, ^15^, ^16^, ^17^, ^18^, ^19^ The potential mechanisms underlying these poor outcomes are the uninflamed phenotype of the microenvironment and the low immunogenicity of *EGFR*‐mutant lung cancer.^20^, ^21^, ^22^ Preclinical trials suggest that chemotherapy or antiangiogenic therapy plus immunotherapy has synergistic effects by reducing the percentage of tumor‐infiltrating regulatory T cells and mediating the tumor microenvironment.^23^, ^24^, ^25^ Promising data from the IMpower150 study has inspired the combination of chemotherapy, bevacizumab and immune checkpoint inhibitors (ICIs) as a salvage option for this patient group.^26^, ^27^ Other clinical trials of combination immunotherapy, such as Keynote 789 and Checkmate 722, are ongoing. Therefore, the current data on the combination of chemotherapy or antiangiogenic drugs and ICIs in such patients are limited.

Pending the outcomes of prospective clinical studies and proven therapeutic strategies in this setting, attention must turn toward the potential applicability of currently licensed drugs and any available supporting evidence in real‐world clinical practice. Therefore, this study aimed to investigate the outcomes of immunotherapy in pretreated and advanced NSCLC patients with *EGFR* mutations in the real‐world setting.

## Methods

### Patient data collection

This retrospective study included pretreated and advanced *EGFR* mutant NSCLC patients who received immunotherapy with or without other regimens (chemotherapy or antiangiogenic drugs) from September 2018 to June 2020 at the National Cancer Center/National Clinical Research Center for Cancer/Cancer Hospital. Pretreated and advanced EGFR‐mutated NSCLC patients were defined as failure to respond to EGFR‐TKIs and/or other systemic regimens. Three patients were excluded because they received combination immunotherapy as their first‐line regimen before *EGFR* gene mutations were detected. One patient was excluded for an unknown regimen (Figure S1 in Appendix S1). This study was approved by the ethics committee of the National Cancer Center/National Clinical Research Center for Cancer/Cancer Hospital.

The following demographics, clinical characteristics and treatment information were collected from medical records: sex, age at the primary diagnosis, smoking status, *EGFR* mutations, *EGFR T790M* status after resistance to the first‐line EGFR‐TKIs, PD‐L1 status, Eastern Cooperative Oncology Group performance status (ECOG PS), tumor stage according to the eighth edition of the American Joint Committee on Cancer Tumor‐Node‐Metastasis (AJCC‐TNM) staging system, brain and liver metastasis at immunotherapy onset; local therapy including radiotherapy and surgical operation, chemotherapy usage, targeted therapy and antiangiogenic drug usage, and number of prior systemic regimens before immunotherapy.

In general, imaging examinations at baseline included computed tomography (CT) images of the chest and abdomen, brain magnetic resonance imaging (MRI) and whole bone scans. Treatment efficacy evaluation was undertaken by CT of the chest and abdomen every two or three cycles during treatment. Brain MRI was also performed if deemed necessary. The response to therapies was evaluated as: complete response (CR); partial response (PR); stable disease (SD); and progressive disease (PD) according to the Response Evaluation Criteria in Solid Tumors (RECIST) version 1.1. Progression‐free survival (PFS) was defined as the period from the initiation of immunotherapy to the date of disease progression. The overall response rate (ORR) to ICIs was calculated as the percentage of patients achieving CR and PR on the basis of RECIST 1.1. The disease control rate (DCR) was defined as the proportion of patients achieving CR, PR, or SD based on the RECIST 1.1.

### Molecular diagnostics

*EGFR* gene mutations were detected before the initiation of first‐ or second‐line EGFR‐TKI therapy and confirmed by next‐generation sequencing (NGS) technology or polymerase chain reaction (PCR). Testing specimens originated from the lung (*n* = 13), lymph nodes (*n* = 5), brain metastases (*n* = 2), blood (*n* = 8), pleural fluid (*n* = 1) and uncertain origin (*n* = 2). The *EGFR T790M* status was detected when patients failed to respond to first‐ or second‐line EGFR‐TKIs. PD‐L1 expression was locally measured by immunohistochemistry.

### Statistical analysis

Baseline characteristics in this study were described by applying descriptive statistics. All calculations included calculation of the means ± 95% confidence intervals (95% CIs). Univariate analysis was performed, and survival was assessed using the Kaplan‐Meier log‐rank test. Proportions were compared between groups using the chi‐squared test, and when pertinent, a two‐sided Fisher's test was conducted. Follow‐up visits continued until 17 August 2020. All statistical analyses were performed using SPSS version 20.0 (IBM Corp, Armonk, NY). Tests were two‐sided, and a *P*‐value < 0.05 was considered statistically significant.

## Results

### Patient characteristics

A total of 31 patients were enrolled into this study (Table 1). All patients had adenocarcinoma, and received anti‐programmed death‐1 (anti‐PD‐1) inhibitors (Table S1). The median (range) age of patients at primary diagnosis was 53 (31–83) years. A total of 19 of 31 (61.3%) patients were female. The performance status (PS) ranged from 0 to 2, with 32.3% of patients having a poor performance status (PS = 2) prior to the initiation of immunotherapy, and 23 of 31 (74.2%) patients were never smokers. Of these patients, 23 patients (74.2%) were diagnosed with stage IVc disease, 18 (41.9%) patients had brain metastasis, and seven (22.6%) patients had liver metastasis. Genetic testing identified *EGFR 19del* in 16 (51.6%) patients, *EGFR 21L858R* in 12 (38.7%) patients and other *EGFR* mutations (*S768I*/*G719X*/unknown) in three (9.7%) patients. Tumors had a positive *EGFR T790M* status in eleven (35.5%) patients, and the PD‐L1 status was positive in seven (22.6%) patients.

**Table 1:** Baseline characteristics of advanced NSCLC patients with *EGFR* mutations

Character	N (%)
Age, year	
Median (range)	53 (31–83)
Gender	
Female	19 (61.3%)
Male	12 (38.7%)
ECOG PS	
0	4 (12.9%)
1	17 (54.8%)
2	10 (32.3%)
Smoking status	
Never	23 (74.2%)
Ever	8 (25.8%)
Pathology	
Adenocarcinoma	31 (100%)
Tumor stage	
IVA	5 (16.1%)
IVB	3 (9.7%)
IVC	23 (74.2%)
Metastatic sites	
Number (median, range)	3 (1–5)
Lymph nodes	18 (58.1%)
Brain	18 (41.9%)
Lung	17 (54.8%)
Bone	16 (51.6%)
Liver	7 (22.6%)
Pleura	11 (35.5%)
Adrenal glands	4 (12.9%)
*EGFR* gene mutation	
*EGFR 19del*	16 (51.6%)
*EGFR 21L858R*	12 (38.7%)
Other	3 (9.7%)
*EGFR T790M* status	
Positive	11 (35.5%)
Negative	15 (48.4%)
Not detected	5 (16.1%)
PD‐L1 status	
≥50%	5 (16.1%)
1–50%	2 (6.5%)
Negative/unknown	24 (77.4%)

The line number of systemic regimens ranged from 1 to 9, with a median line number of four, prior to the initiation of ICIs. All patients received targeted therapy. More than half of patients received chemotherapy (74.2%, 23/31) or antiangiogenic drugs (74.2%, 23/31) before ICIs. A total of 11 (35.5%) patients received radiotherapy or surgery before ICIs. Six patients (19.4%) were treated with immunotherapy as a single agent. Approximately 25 of 31 (80.6%) patients received combination immunotherapy with other agents: osimertinib (1, 3.2%); chemotherapy (9, 29.0%); antiangiogenic drugs (9, 29.0%); chemotherapy and antiangiogenic drugs (6, 19.4%) (Table 2).

**Table 2:** Immunotherapy therapy and clinical outcomes of advanced NSCLC patients with *EGFR* mutations

Character	N (%)
Number of prior systemic regimens	
Median (range)	4 (1–9)
Prior systemic therapy	
Targeted therapy	31 (100%)
Chemotherapy	
No	8 (25.8%)
Yes	23 (74.2%)
Antiangiogenic therapy	
No	8 (25.8%)
Yes	23 (74.2%)
Local therapy	
No	20 (64.2%)
Yes	11 (35.5%)
Immunotherapy regimens	
Combination therapy	25 (80.6%)
ICIs plus osimertinib	1 (3.2%)
ICIs plus chemotherapy	9 (29.0%)
ICIs plus antiangiogenic drugs	9 (29.0%)
ICIs plus chemotherapy and antiangiogenic drugs	6 (19.4%)
Monotherapy	6 (19.4%)

### Univariate and multivariate analysis

**Figure 1:**
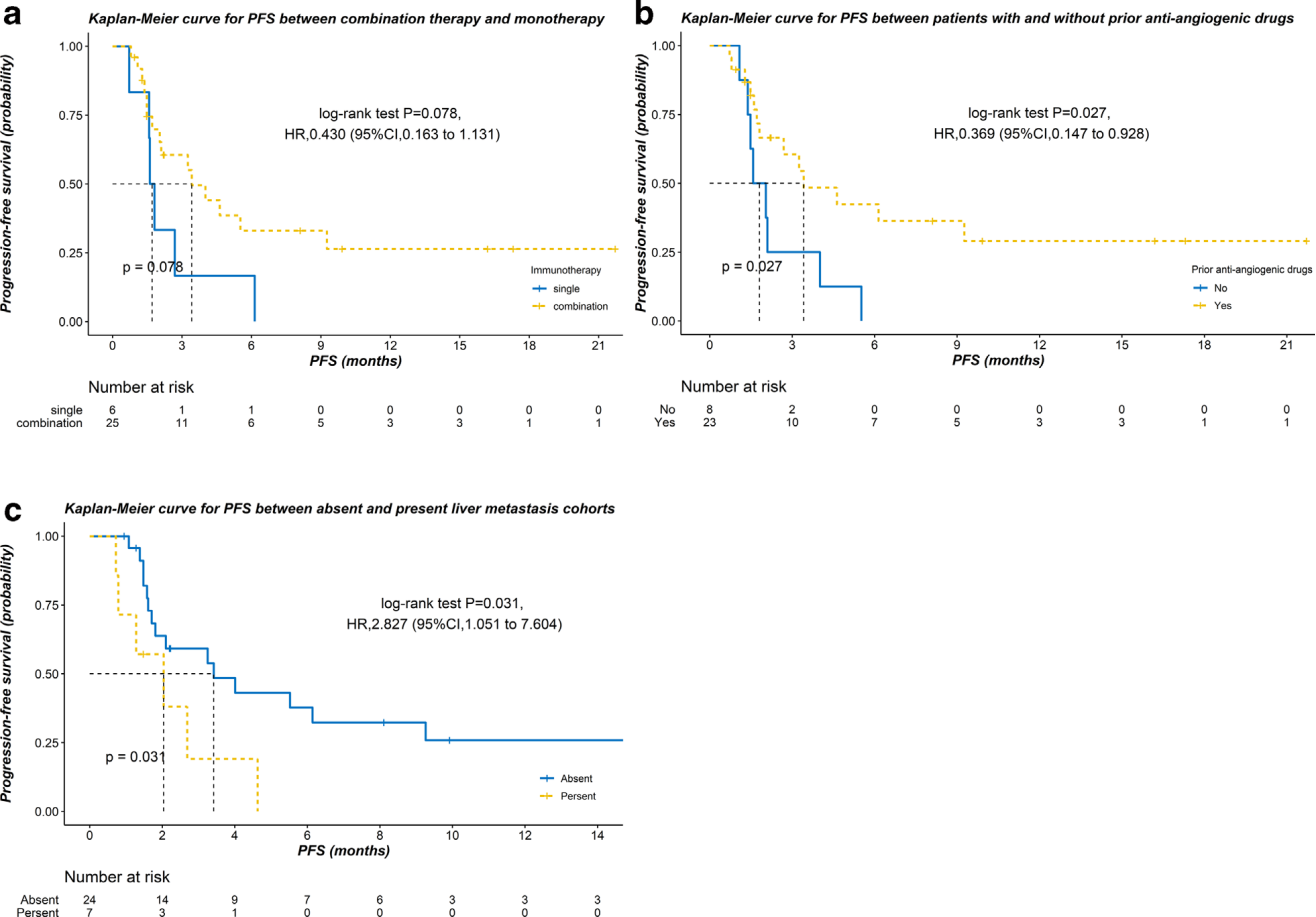
The Kaplan‐Meier curve for progression‐free survival (PFS) in terms of (**a**) immunotherapy regimens (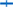) single and (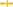) combination; (**b**) prior antiangiogenic drugs therapy; (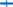) No and (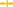) Yes and (**c**) liver metastasis (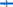) Absent and (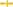) Present.

The median follow‐up time was 11.24 (95% CI: 8.69–13.78) months. The median PFS (mPFS) was 3.25 (95% CI: 1.65–4.86) months, and the median overall‐survival (OS) was not reached. In the univariate analysis, patients who received combination immunotherapy therapy seemingly acquired longer PFS than those who received immunotherapy as a single agent, although there was no significant difference in terms of PFS (3.42 vs. 1.61 months; *P* = 0.078; hazard ratio (HR) 0.43, 95% CI: 0.16–1.13) (Fig 1a). Patients who received antiangiogenic drugs prior to immunotherapy acquired better PFS than those did not (3.42 vs. 1.58 months; *P* = 0.027; HR 0.37, 95% CI: 0.15–0.93) (Fig 1b). However, patients with liver metastasis obtained inferior PFS (2.04 vs. 3.42 months; *P* = 0.031; HR 2.83, 95% CI: 1.05–7.60) (Fig 1c). In addition, multivariate analysis confirmed that immune‐based combination regimens, prior antiangiogenic drugs and liver metastasis were independent prognostic factors of PFS in this setting (Table 3).

**Table 3:** Univariate and multivariate analysis

		Univariate analysis		Multivariate analysis	
Covariates	Comparisons	Median PFS in months (95% CI)	*P*‐value	*P*‐value	HR (95% CI)
Gender	Male vs. female	1.70 (0.00–4.94) vs. 3.25 (1.65–4.86)	0.820		
ECOG PS	1 vs. 2	3.25 (0.00–6.61) vs. 1.81 (0.22–3.40)	0.225		
Smoking status	Never vs. ever	2.69 (0.99–4.40) vs. NR	0.168		
Tumor stage	IVB vs. IVA	1.38 (0.91–1.85) vs. 2.69 (1.42–3.96)	0.452		
	IVC vs. IVA	3.42 (0.92–5.92) vs. 2.69 (1.42–3.96)	0.857		
Brain metastasis	Present vs. absent	2.69 (1.00–4.39) vs. 4.63 (0.45–8.82)	0.397		
Liver metastasis	Present vs. absent	2.04 (0.49–3.59) vs. 3.42 (0.88–5.96)	**0.031**	**0.041**	**2.96 (1.05–8.34)**
*EGFR* gene mutation	EGFR L858R vs. EGFR 19del	5.52 (0.00–11.24) vs. 2.04 (1.32–2.76)	0.284		
EGFR T790M status	Positive vs. negative	2.69 (1.20–4.19) vs. 3.42 (0.98–5.85)	0.893		
PD‐L1 status	1%–50% vs. ≥50%	NA vs. 6.14 (2.19–4.63)	0.771		
	Negative/unknown vs. ≥ 50%	2.69 (0.76–4.63) vs. 6.14 (2.19–10.10)	0.348		
Local therapy	Yes vs. no	1.58 (1.33–1.83) vs. 2.10 (0.00–4.47)	0.762		
Prior chemotherapy	Yes vs. no	3.25 (1.22–5.28) vs. 2.10 (1.93–2.27)	0.736		
Prior antiangiogenic drugs therapy	Yes vs. no	3.42 (0.97–5.87) vs. 1.58 (0.80–2.35)	**0.027**	**0.023**	**0.27 (0.09–0.83)**
Immunotherapy regimens	Combination therapy vs. monotherapy	3.42 (1.94–4.90) vs. 1.61 (1.33–1.89)	**0.078**	**0.044**	**0.346 (0.12–0.97)**

### Immunotherapy regimens and clinical outcomes

**Figure 2:**
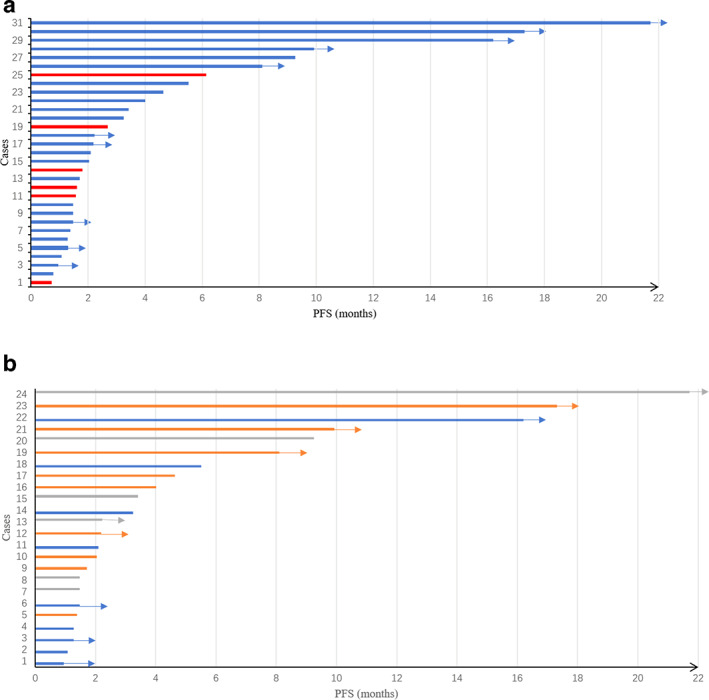
The swimmer plot of progression‐free survival (PFS) from the initiation of immunotherapy according to (**a**) the entire cohort; (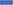) Combination therapy and (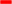) monotherapy and (**b**) different combination regimens (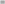) ICIs plus chemotherapy and antiangiogenic drugs, () ICIs plus chemotherapy, and () ICIs plus antiangiogenic drugs.

**Figure 3:**
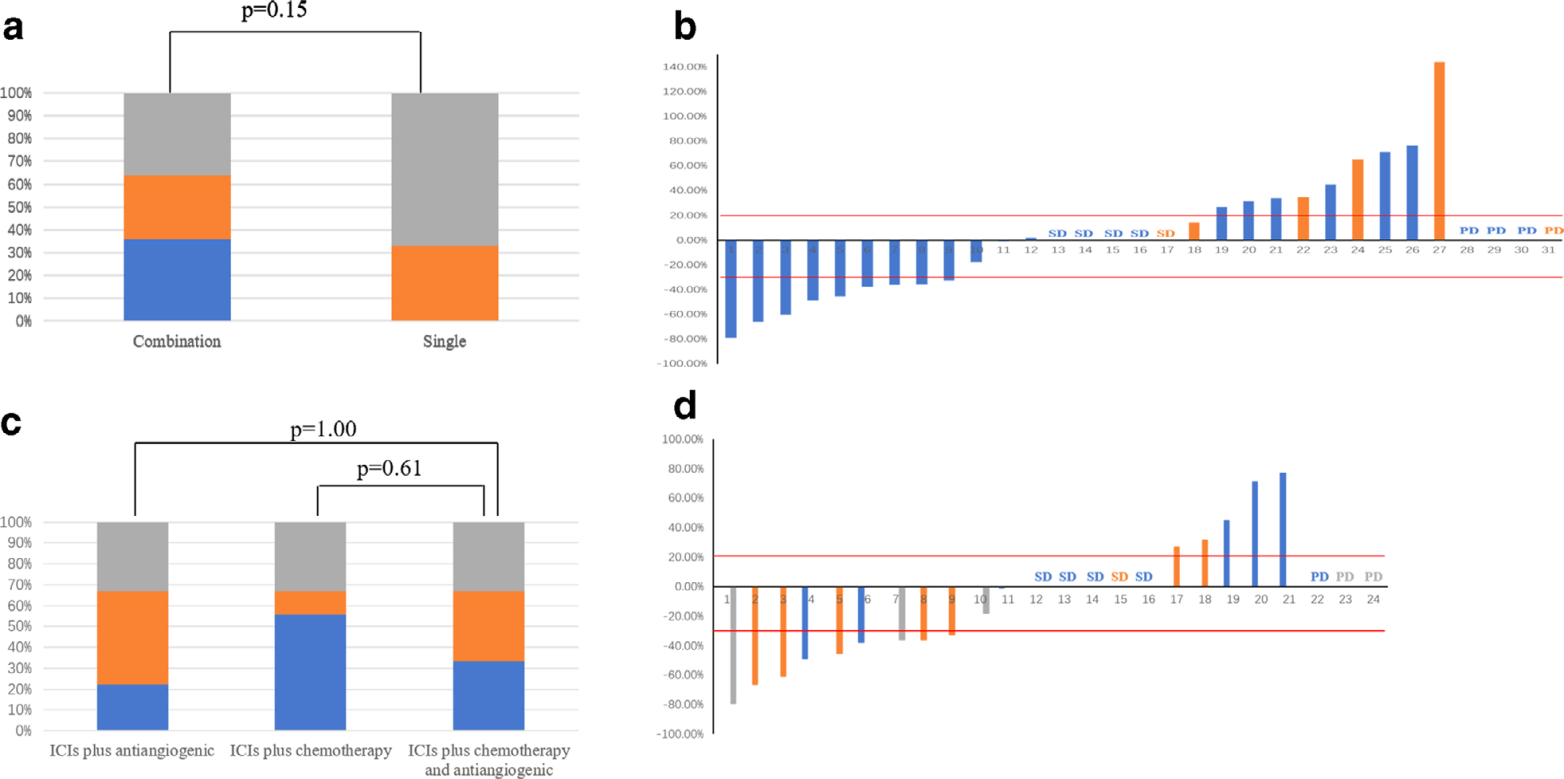
The best response of enrolled patients received immunotherapy. (**a**) The overall response rate (ORR) was not significant different between immunotherapy as single regimens and combination regimens () PR, () SD, and () PD. (**b**) The best response rate of all enrolled patients with immunotherapy is illustrated () combination therapy, and () monotherapy. (**c**) The ORR was not statistically different among three different combination immunotherapy regimens. (**d**) The best response rate of all enrolled patients with combination immunotherapy is illustrated () ICIs plus antiangiogenic, () ICIs plus chemotherapy, and () ICIs plus chemotherapy and antiangiogenic drugs.

The swimmer plot of different therapeutic strategies in this setting is shown in Fig 2. No patients achieved partial response in the monotherapy cohort, and the DCR was 2/6 in this group. Among the combination groups, the ORR was 36.0% and the DCR was 64.0%. There was no significant difference between ICI monotherapy and immune‐based combination therapy in terms of ORR (*P* = 0.15) (Fig 3a,b). In the ICIs plus chemotherapy cohort, the ORR and the DCR was 5/9 and 6/9, respectively. In the ICIs plus antiangiogenic drugs group, the ORR and the DCR was 2/9 and 6/9, respectively. The ORR was 2/6 and the DCR was 4/6 in patients receiving ICIs plus chemotherapy and antiangiogenic drugs. No significant difference was analyzed among three different combination strategies in terms of ORR (ICIs plus antiangiogenic drugs vs. ICIs plus chemotherapy and antiangiogenic inhibitors, *P* = 1.00; ICIs plus chemotherapy vs. ICIs plus chemotherapy and antiangiogenic inhibitors, *P* = 0.61; Fig 3c,d).

### Intracranial response to ICIs

There were 18 patients diagnosed with brain metastasis prior to the initiation of ICIs. Three and 15 patients received single agent and combination therapies, respectively. The best intracranial response to ICIs was SD. The DCR in terms of intracranial response was in two of three patients who had received immunotherapy as a single agent and 60% (9/15) in the combination group, respectively.

### Safety

Four patients (12.9%) experienced grade 1 to grade 2 immune‐related adverse events, including one patient receiving single‐agent therapy who had a grade 1 rash, one patient who received ICI combination therapy and antiangiogenic drugs who had grade 2 hypothyroidism, and two patients receiving combination immunotherapy with chemotherapy who had a grade 2 rash.

## Discussion

This retrospective study provided evidence on the efficacy of immunotherapies in advanced *EGFR*‐mutated NSCLC patients who failed to respond to EGFR‐TKIs and multiple conventional systemic therapies in a real‐world setting. Immunotherapy‐based combination therapy was a favorable prognostic factor, and furthermore, in addition to the results of previous studies, this study showed the PFS benefit of anti‐PD‐1

inhibitors when

combined with other agents.

ICI monotherapy has been reported to have a reduced efficacy in heavily treated *EGFR* mutant NSCLC patients who have failed to respond to EGFR‐TKIs. However, the efficacy of immunotherapy combination therapy has been under debate until now. Previous studies have demonstrated that the PFS of ICI monotherapy in patients ranged from 1.6 to 1.9 months in this population,^19^, ^28^ which is similar to the finding in our monotherapy cohort. Furthermore, using ICI monotherapy in patients with *EGFR* mutant NSCLC who failed to respond to EGFR‐TKIs and without acquired *T790M* mutation is not recommended in the clinical practice guidelines of the European Society for Medical Oncology (ESMO).^29^ However, rare data on ICI‐based combination therapies has been reported and the guidelines regarding combined immunotherapy in this setting remains unclear. Previous mainstream systematic regimen in this setting was conventional chemotherapy. In the AURA3 study, the median PFS and ORR of the platinum plus pemetrexed group was reported to be 4.4 months and 31%, respectively.^30^ Although there were more heavily‐treated patients in our study, the median PFS of immunotherapy‐based combination therapy in our study was comparable to the efficacy of platinum‐based chemotherapy in the AURA3 study. The ABCP (atezolizumab plus platinum‐based chemotherapy and bevacizumab) arm in the IMpower150 study is the first and only reported example of a survival benefit for ICI‐based combination therapy in *EGFR*‐mutant NSCLC patients who failed to respond to prior treatment with TKIs, achieving an ORR of 72% and a median OS of 19.4 months.^27^ The ORR of 33.3% (2/6) in a four‐drug combination regimen in our study was obviously lower than that of previously reported data. The difference might be explained by the limited population size and relatively heavily treated patients in this study. The synergistic activities of immunotherapy with chemotherapy or antiangiogenic agents have been confirmed in preclinical studies,^31^ and relevant prospective clinical trials; for example Keynote 789 (NCT03515837), and Checkmate 722 (NCT02864251), are presently under investigation. In the meantime, several immunotherapy‐based combination regimens illustrated in our study have been utilized in the real‐world. Qiu *et al*. recently reported a median PFS of 5.4 months in *EGFR*‐mutant patients who received ICIs plus antiangiogenic drugs; however they did not provide a specific description of this setting.^32^ This study, which complements existing studies, highlighted that immunotherapy, especially anti‐PD‐1, combined with different agents benefited pretreated *EGFR*‐mutated NSCLC patients in a real‐world setting.

Our study also found that patients with liver metastasis had an inferior PFS. The underlying mechanism might be explained by the highly immunosuppressive liver microenvironment.^33^, ^34^ In addition, several studies have previously reported shorter OS in advanced NSCLC patients with liver metastasis than those without it, while receiving ICI as a single agent.^27^, ^35^, ^36^, ^37^, ^38^ Although many prospective clinical studies enrolled some patients with liver metastasis, few studies have referred to the efficacy of immunotherapy combination regimens in advanced lung cancer patients with liver metastasis. The subgroup analysis in the IMpower150 study demonstrated that improved median OS with ABCP regimens was observed in patients with liver metastasis.^27^ Considering the poor prognosis, patients with liver metastasis might need more aggressive and combined therapy to control their disease. This study revealed that patients with antiangiogenic drugs prior to immunotherapy obtained PFS benefit. Although few studies have previously reported this phenomenon, it has been speculated that tumor vascular normalization after use of antiangiogenic agents might potentially enhance the efficacy of subsequent treatments.^39^

Some limitations in this study should be considered. First, heterogenous treatment options in this study were due to its retrospective nature and limited sample size, and therefore the results should be interpreted with caution. Second, the median overall survival was not reached, as we were unable to conclude whether combined immunotherapy would lead to long‐term survival. In addition, some details regarding the detection kit used to evaluate PD‐L1 expression were unavailable. We failed to detect the *EGFR T790M* gene beyond first‐ or second‐line EGFR‐TKI resistance in a small number of patients due to unavailable or inadequate tissue biopsy specimens.

In conclusion, the findings of our study indicated that ICI‐based combination therapies might be more beneficial than ICI monotherapy in pretreated and *EGFR*‐mutant NSCLC patients with progressive disease. This study provided more evidence on anti‐PD‐1 inhibitors in combination with different regimens in this setting. In addition, prior antiangiogenic drugs before immunotherapy might be a favorable prognostic factor, while liver metastasis was associated with a short PFS in such patients. More robust clinical trial results should be performed in order to guide clinical practice in the future.

## Disclosure

The authors declare that the research was conducted in the absence of any commercial or financial relationships that could be construed as a potential conflict of interest.

## Supporting information


**Appendix S1**: Supporting information.
