# Supplementary material for: Superior efficacy of immunotherapy‐based combinations over monotherapy for EGFR‐mutant non‐small cell lung cancer acquired resistance to EGFR‐TKIs
Source: Thorac Cancer. 2020 Oct 19;11(12):3501–9. doi: 10.1111/1759-7714.13689 (PMC7705617; doi:10.1111/1759-7714.13689)
Supplement: Supplementary file 1 — Appendix S1: Supporting information. [file TCA-11-3501-s001.docx]

**Supplemental figure1 Flow chart**


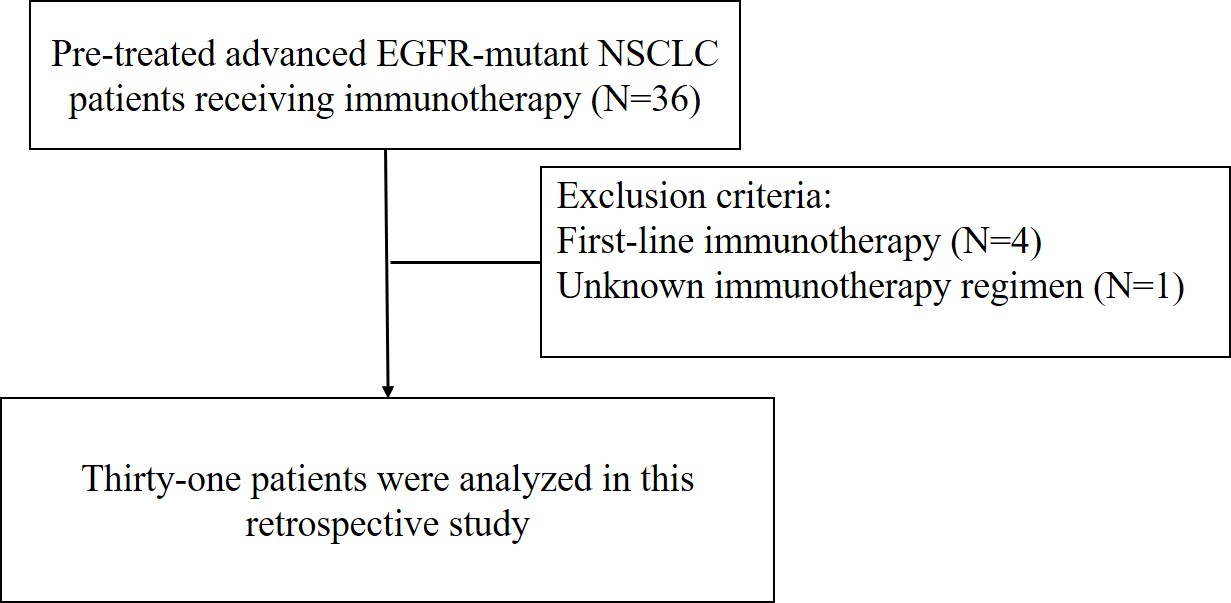


Supplemental Table 1

| Name of anti-PD-1 inhibitors | Number (%) |
| --- | --- |
| Pembrolizumab | 9 (29.0%) |
| Nivolizumab | 3 (9.7%) |
| Camrelizumab | 1 (3.2%) |
| Toripalimab | 6 (19.4%) |
| Sintilimab | 11 (35.5%) |
| Unknown* | 1 (3.2%) |

*This patient was enrolled into one prospective clinical trial.
